# Dose–Response and survival analysis after transarterial radioembolization for intrahepatic cholangiocarcinoma: A multicenter multicompartment dosimetry study

**DOI:** 10.1007/s00259-026-08007-w

**Published:** 2026-06-23

**Authors:** Cigdem Soydal, Tunc Ones, Murat Fani Bozkurt, Emine Goknur Isık, Nalan Alan Selcuk, Nazım Coskun, Ramazan Kutlu, Burcu Esen Akkas, Hasan Onner, Aziz Gultekin, Semra Ince, Pınar Ozbay, Seyhan Karacavus, Irem Mesci, Efe Soydemir, Bilge Volkan Salancı, Güney Deniz, Osman Melih Topcuoğlu, Elif Ozdemir, Müge Otlu, Enes Mustafa Kaya, Alaaddin Nayman, Muhammet Arslan, Hulya Peker, Huseyin Tugsan Ballı, Mustafa Bilgili, Emre Can Celebioglu, Erbil Arik, Gonca Eldem, M.D, Arzu Poyanli, Türkay Toklu, Mustafa Ozdemir, Meryem Kaya, Ismail Dilek, Ali Celik, Gökhan Yuce, Fadime Demir, Nuriye Ozlem Kucuk

**Affiliations:** 1 Medical School Department of Nuclear Medicine, Ankara University, 06590 Cebeci Ankara, Türkiye; 2 Department of Nuclear Medicine, Marmara University Pendik Training and Research Hospital, Istanbul, Türkiye; 3 Medical School Department of Nuclear Medicine, Hacettepe University, Ankara, Türkiye; 4 Medical School Department of Nuclear Medicine, Istanbul University, Istanbul, Türkiye; 5 Medical School Department of Nuclear Medicine, Yeditepe University, Istanbul, Türkiye; 6 Ankara Bilkent City Hospital Department of Nuclear Medicine, Ankara, Türkiye; 7 Medical School Department of Radiology, Inonu University, Malatya, Türkiye; 8 Basaksehir Cam Ve Sakura City Hospital Department of Nuclear Medicine, Istanbul, Türkiye; 9 Medical School Department of Nuclear Medicine, Selcuk University, Konya, Türkiye; 10 Medical School Department of Nuclear Medicine, Pamukkale University, Denizli, Türkiye; 11 Department of Nuclear Medicine, Gulhane Training and Research Hospital, Ankara, Türkiye; 12 Department of Nuclear Medicine, Acıbadem Adana Hospital, Adana, Türkiye; 13 Department of Nuclear Medicine, Health Sciences University Kayseri City Health Practice and Research Hospitals, Kayseri, Türkiye; 14 Department of Radiology, Marmara University Pendik Training and Research Hospital, Istanbul, Türkiye; 15 Medical School Department of Radiology, Yeditepe University, Istanbul, Türkiye; 16 Department of Nuclear Medicine, Malatya Training and Research Hospital, Malatya, Türkiye; 17 Medical School Department of Radiology, Selcuk University, Konya, Türkiye; 18 Medical School Department of Radiology, Pamukkale University, Denizli, Türkiye; 19 Department of Radiology, Acıbadem Adana Hospital, Adana, Türkiye; 20 Department of Radiology, Health Sciences University Kayseri Health Practice and Research Hospitals, Kayseri, Türkiye; 21 Medical School Department of Radiology, Ankara University, Ankara, Türkiye; 22 Medical School Department of Radiology, Hacettepe University, Ankara, Türkiye; 23 Medical School Department of Radiology, Istanbul University, Istanbul, Türkiye; 24 Ankara Bilkent City Hospital Department of Radiology, Ankara, Türkiye; 25 Department of Radiology, Gulhane Training and Research Hospital, Ankara, Türkiye

**Keywords:** Cholangiocarcinoma, Transarterial radioembolization, Y-90 microspheres, Multicompartment dosimetry, Treatment response

## Abstract

**Aim:**

We aimed to report real-world outcomes of transarterial radioembolization (TARE) in patients with intrahepatic cholangiocarcinoma (ICC), focusing on different microsphere types and posttreatment dosimetric approaches in relation to dose–response and survival analyses.

**Materials and methods:**

This multicenter retrospective single-arm cohort study included adult patients with intrahepatic cholangiocarcinoma treated with Y-90 glass or resin microspheres using lobar or segmental approaches between January 2014 and December 2024 across 13 centers. All posttreatment Y-90 Bremsstrahlung single photon emission tomography (SPECT)/ computed tomography (CT) or Y-90 positron emission tomography (PET) images were reviewed. Post-treatment dose estimations were performed centrally by a nuclear medicine physician with 10 years of experience in dosimetric calculations using the VoxelDosimetry tool of Hermia software (Hermes Medical Solutions, Sweden). Mean perfused liver absorbed dose (PLAD), mean tumor absorbed dose (TAD), and mean whole-liver absorbed dose (WLAD) were calculated. Dosimetric analysis for Y-90 SPECT/CT and PET/CT were performed separately. Tumor response was assessed by comparing imaging obtained within 6 weeks before treatment and 2–4 months after treatment. Response categories were grouped as objective response (complete response + partial response) and nonresponse (stable disease + progressive disease) for treated lesions. Hepatotoxicity was graded according to the Common Terminology Criteria for Adverse Events (CTCAE) using serum Aspartate Aminotransferase (AST), Alanine Aminotransferase (ALT), and bilirubin levels within 3 months after TARE. Additionally, albumin–bilirubin (ALBI) scores were calculated before and after treatment, and changes in ALBI grade were analyzed.

**Results:**

Initially, data from 194 (110 female, 84 male; mean age 53.8 ± 16.6 years) patients were included. After excluding patients without survival status data, 180 patients were included in the overall survival (OS) analysis. Seventeen patients were excluded from dose–response evaluation due to suboptimal posttreatment Y-90 imaging for dosimetric calculations. Survival analyses were performed on a per-patient basis, whereas dosimetric and response analyses were performed on a per-treatment-session basis. For dose response analysis, a total of 194 TARE sessions in 163 (101 female, 62 male; mean age 51.4 ± 12.6 years) patients were analyzed. Median (min–max) of administered activities were 2.7 (1.4–6.1) GBq for glass microspheres and 1.4 (0.6–1.8) GBq for resin microspheres (p = 0.004). In separate analyses of SPECT and PET studies, higher TAD and PLAD values were observed in sessions analyzed with PET-based dosimetry (for mean TAD; 249.2 ± 26.6 Gy vs 114.8 ± 15.7 Gy, p < 0.001, for mean PLAD; 102.1 ± 10.4 Gy vs 59.4 ± 11.3 Gy, p = 0.031). A significant positive trend between TAD and response category was observed in the resin microsphere sessions using Y-90 PET–based dosimetry (Area Under Curve 0.693, p = 0.028). In the OS analysis of 180 patients (median follow-up 13.3 months, range 7–81), 128 deaths occurred. Medians of OS did not differ between patients treated with glass versus resin microspheres [17.4 (13.2–21.5, 95%CI) vs 16.6 (7.7–25.5, 95%CI) months, p = 0.80]. Among 107 patients included in time to progression (TTP) analysis, no significant difference in medians of TTP was observed between patients treated with glass and resin microspheres [12.1 (7.8–12.4, 95%CI) vs 14.5 (5.6- 14.4, 95%CI) months, p = 0.11]. One patient developed grade 2 hepatotoxicity and irreversible hepatic failure 10 months after a second TARE session due to tumor progression. In addition, cholangitis occurred in 1 patient and gastritis in 3 patients following TARE.

**Conclusion:**

This multicenter analysis suggests that TARE may provide encouraging survival outcomes with acceptable toxicity in patients with ICC, including salvage settings. A potential dose–response association was identified in the resin microsphere subgroup evaluated with Y-90 PET-based dosimetry. Given the retrospective multicenter design and heterogeneity of imaging modalities, these findings should be considered hypothesis-generating and require confirmation in prospective studies using standardized posttreatment dosimetry.

## Introduction

Intrahepatic cholangiocarcinoma (ICC) is a primary liver malignancy that remains challenging to manage because of its aggressive biology and limited therapeutic options [1–4]. The incidence of ICC has been increasing, partly due to its frequent association with underlying chronic liver disease [5]. Surgical resection remains the only potentially curative treatment; however, because of advanced disease stage or comorbidities, fewer than 30% of patients are candidates for surgery at the time of diagnosis [6].

In contemporary oncologic practice, gemcitabine plus cisplatin combined with durvalumab has become the standard first-line systemic therapy based on the results of the TOPAZ-1 trial. Although the addition of durvalumab to chemotherapy demonstrated a statistically significant improvement in overall survival compared with chemotherapy alone, the magnitude of benefit was modest (median overall survival 12.9 vs 11.3 months) [7, 8].

Transarterial radioembolization (TARE) has been increasingly used for the treatment of primary and secondary liver tumors because it enables delivery of high absorbed doses to tumors while minimizing exposure to normal liver parenchyma. Its safety and therapeutic effectiveness have improved with the implementation of multicompartment dosimetric approaches [9, 10]. Although phase II studies have demonstrated the safety and feasibility of combining TARE with chemotherapy as a first-line treatment strategy, in routine clinical practice most patients receive TARE after failure of gemcitabine- and cisplatin-based chemotherapy [11].

In this multicenter retrospective cohort study, we report real-world outcomes of TARE in patients with ICC, focusing on different microsphere types and posttreatment dosimetric approaches in relation to dose–response relationships and survival outcomes.

## Materials and methods

### Study cohort

This multicenter retrospective single-arm cohort study included adult patients with intrahepatic cholangiocarcinoma (ICC) treated with Y-90 glass or resin microspheres using lobar or segmental approaches between January 2014 and December 2024 across 13 centers. A written informed consent was obtained from all the participants and institutional ethical approval was obtained from the Human Research Ethics Committee of Ankara University (approval no: i11-1039–25 date:23.12.2025). All the data evaluation was performed in accordance with the Declaration of Helsinki.

Exclusion criteria were:

(1) absence of baseline imaging (F-18 fluorodeoxyglucose [FDG] PET/CT, contrast enhanced CT, or liver MRI) within 6 weeks prior to radioembolization;

(2) absence of follow-up imaging (F-18 FDG PET/CT) 2–4 months after radioembolization;

(3) lack of posttreatment Y-90 Bremsstrahlung SPECT/CT or Y-90 PET imaging; or.

(4) suboptimal image quality precluding reliable dosimetric analysis.

### Dosimetry

All posttreatment Y-90 Bremsstrahlung SPECT/CT and Y-90 PET/CT images were performed within 24 h after TARE according to institutional protocols; based on the recommendation guideline of EANM for available PET camera of the center [12]. Because of the multicenter retrospective design including different scanner generations and institutional protocols, acquisition and reconstruction parameters were not completely uniform across centers. Pretreatment activity prescriptions were based on multicompartment partition-model dosimetry using Tc-99 m MAA SPECT/CT in accordance with EANM recommendations [12]. Posttreatment dosimetric calculations were performed centrally by a nuclear medicine physician with 10 years of experience in internal dosimetry using the VoxelDosimetry module of Hermia software (Hermes Medical Solutions, Sweden). Y-90 PET images were reconstructed using iterative reconstruction algorithms available on each system, while Bremsstrahlung SPECT reconstructions were centrally processed using the Hermia platform. Partial-volume correction and recovery coefficient–based corrections were not systematically applied because of retrospective multicenter heterogeneity. Lung and liver segmentations were automatically derived from CT components of SPECT/CT or PET/CT datasets. Perfused liver volumes and tumor volumes were delineated using threshold-based segmentation on SPECT or PET images. The following dosimetric parameters were calculated: mean perfused liver absorbed dose (PLAD), mean tumor absorbed dose (TAD), and mean whole-liver absorbed dose (WLAD).

### Response assessment

All patients had at least one baseline and one follow-up F-18 FDG PET/CT or contrast-enhanced liver CT or MRI after each treatment session. Imaging obtained within 6 weeks before treatment and 2–4 months after treatment was compared. Treatment response was assessed metabolically using PERCIST criteria or radiologically using modified RECIST (mRECIST) criteria for only lesions that are treated with TARE. Because the primary aim of the response analysis was to evaluate local dose–response relationships within treated lesions, new lesions outside the treated volume were recorded separately and were not automatically classified as progressive disease. This approach differs from standard RECIST/PERCIST methodology and should be interpreted within the context of lesion-directed dosimetric analysis.

Response categories were grouped as objective response (complete response [CR] + partial response [PR]) and nonresponse (stable disease [SD] + progressive disease [PD]). The occurrence of new lesions after radioembolization was recorded but not classified as PD, as the analysis focused on dose–response relationships in treated lesions only. All assessments were performed on a per-treatment session basis for multivariate analysis of predictive factors of response.

### Dose–toxicity evaluation

Hepatotoxicity was graded according to the Common Terminology Criteria for Adverse Events (CTCAE) using serum Aspartate Aminotransferase (AST), Alanine Aminotransferase (ALT) and bilirubin levels within 3 months after each TARE session. Additionally, albumin–bilirubin (ALBI) scores were calculated before and after treatments, and changes in ALBI grade were analyzed. Other side effects during follow-up periods that can be related to TARE were recorded.

### Statistical analysis

Continuous variables were compared between groups using the independent-samples t-test. Categorical variables were analyzed using the chi-square test. Tumor absorbed dose (TAD) values between responders (CR + PR) and nonresponders (SD + PD) were compared using the Mann–Whitney U test because dosimetric variables were not normally distributed. Receiver operating characteristic (ROC) analysis was used to determine optimal cut-off values of mean TAD for predicting objective response (CR + PR). Area under the curve (AUC) values were calculated for predictive models. Optimal cut-off values were also determined for survival analyses.

TTP was defined as the time from TARE to documented radiological or metabolic progression. Death without documented progression was censored at the date of death or last follow-up.

Survival analyses were performed on a per-patient basis based on the first treatments of patients, whereas dosimetric and response analyses were performed on a per-treatment-session basis. Survival analyses were performed independently from lesion-based response analyses. OS and TTP were estimated using the Kaplan–Meier method. Statistical analyses were performed using IBM SPSS Statistics, version 27.0. A p-value < 0.05 was considered statistically significant.

## Results

**Fig. 1:**
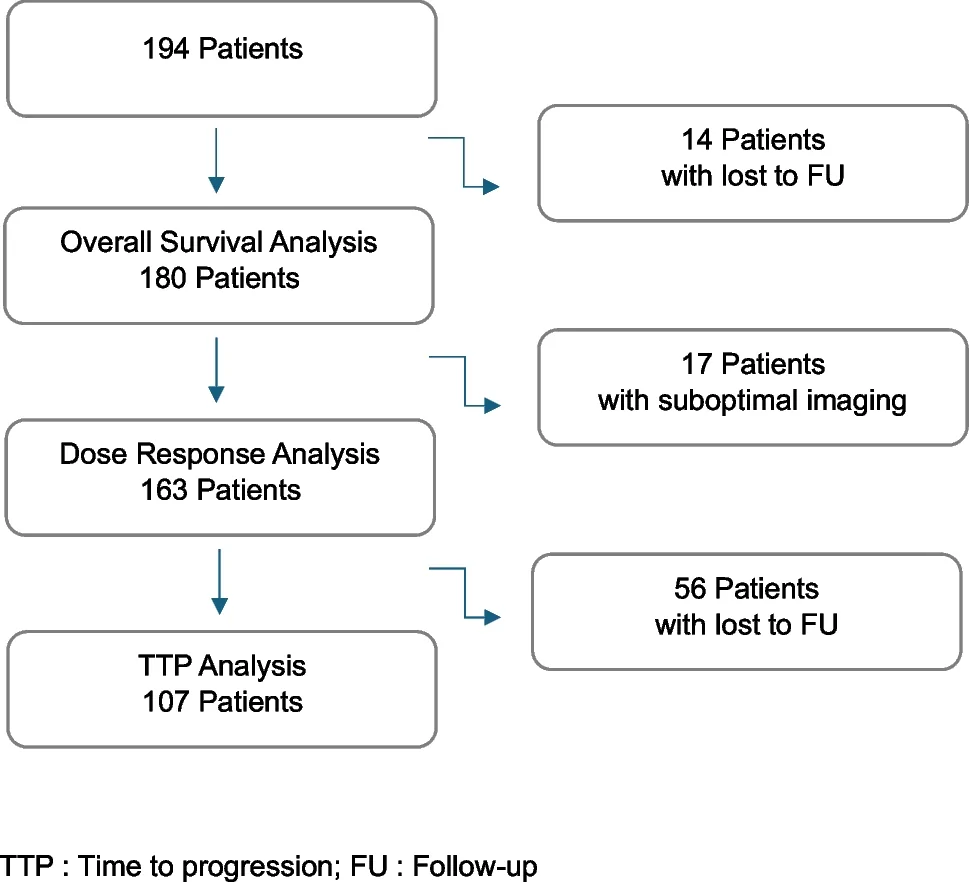
Flowchart of patient inclusion

Data from 194 (110 female, 84 male; mean age 53.8 ± 16.6 years) patients were initially included in the study. After exclusion of patients without available survival status data, 180 patients were included in the OS analysis. Seventeen patients were excluded from the dose–response analysis due to suboptimal posttreatment Y-90 SPECT or PET imaging. In total, 194 TARE sessions performed in 163 patients (101 female, 62 male; mean age 51.4 ± 12.6 years) were included in the final analysis (Fig. 1).

Seventy-eight patients had received systemic chemotherapy prior to TARE, whereas 2 and 3 patients had previously undergone transarterial chemoembolization (TACE) and surgical resection, respectively. Histopathologic subtype data were available for 67 patients, including mass-forming type in 57 and periductal infiltrating type in 10.

Tumor distribution was confined to the right lobe in 90 patients (55%), the left lobe in 67 patients (41%), and was bilobar in 6 patients (4%). A single lesion was treated in 128 sessions (66%), whereas multiple lesions (≥ 2) were treated in the remaining sessions.

TARE was performed using glass and resin microspheres in 107 (55%) and 87 (45%) sessions, respectively. Median (min–max) of administered activities were 2.7 (1.4–6.1) GBq for glass microspheres and 1.4 (0.6–1.8) GBq for resin microspheres (p = 0.004).

### Dose response evaluation

Mean TAD was 233.8 ± 188.3 Gy for resin microsphere sessions and 182.0 ± 54.1 Gy for glass microsphere sessions (p = 0.35). Posttreatment dosimetric analyses were performed using Y-90 PET in 105 sessions (54%) and Y-90 SPECT in 89 sessions (46%). Higher estimated TAD values were observed in sessions analyzed with PET-based dosimetry (249.2 ± 26.6 Gy vs 114.8 ± 15.7 Gy, p < 0.001). A similar difference was observed for mean PLAD between PET and SPECT dosimetry (102.1 ± 10.4 Gy vs 59.4 ± 11.3 Gy, p = 0.031). Details of dosimetric parameters stratified by microsphere type and posttreatment imaging modality are presented in Table 1. Treatment response categories were CR in 39 treatments (20%), PR in 78 (40%), SD in 58 (30%), and PD in 19 (10%).

**Table 1:** Details of dosimetric data based on microspheres types and posttreatment imaging methods. Data are presented as mean ± standard deviation (SD)

	**Mean TAD (Gy)**		**Mean PLAD (Gy)**		**Mean WLAD (Gy)**	
Microspheres Type (n)						
Glass (107)	182.0±54.1	*p=0.35*	81.4±11.8	*p=0.29*	32.7±5.0	p=0.95
Resin (87)	233.8±188.3		93.2±10.2		39.8±7.8	
Posttreatment imaging method (n)						
SPECT (89)	114.7±15.6	*p<0.001*	59.4±11.3	p=0.03	29.2±4.3	p=0.14
PET (105)	249.2±26.6		102.1±10.4		39.7±7.2	

Gy;gray, SPECT; single photon emission tomography, PET; positron emission tomography, n; number

**Fig. 2:**
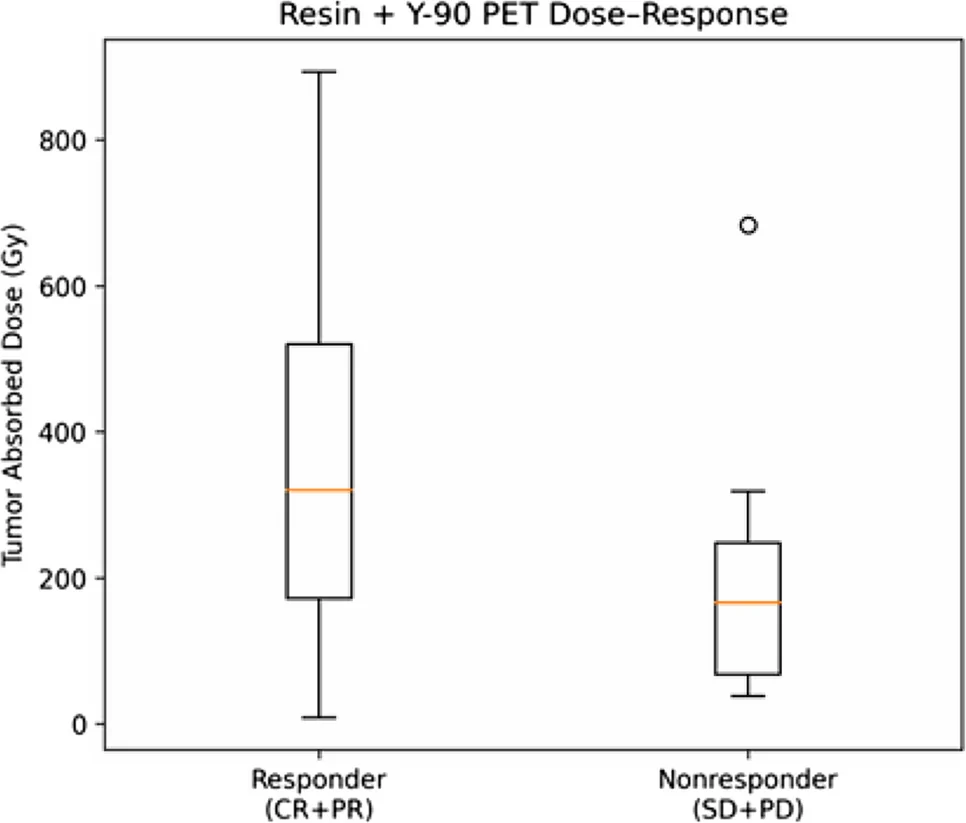
Relationship between tumor absorbed dose and response categories in resin microsphere treatments with Y-90 PET–based dosimetry

**Fig. 3:**
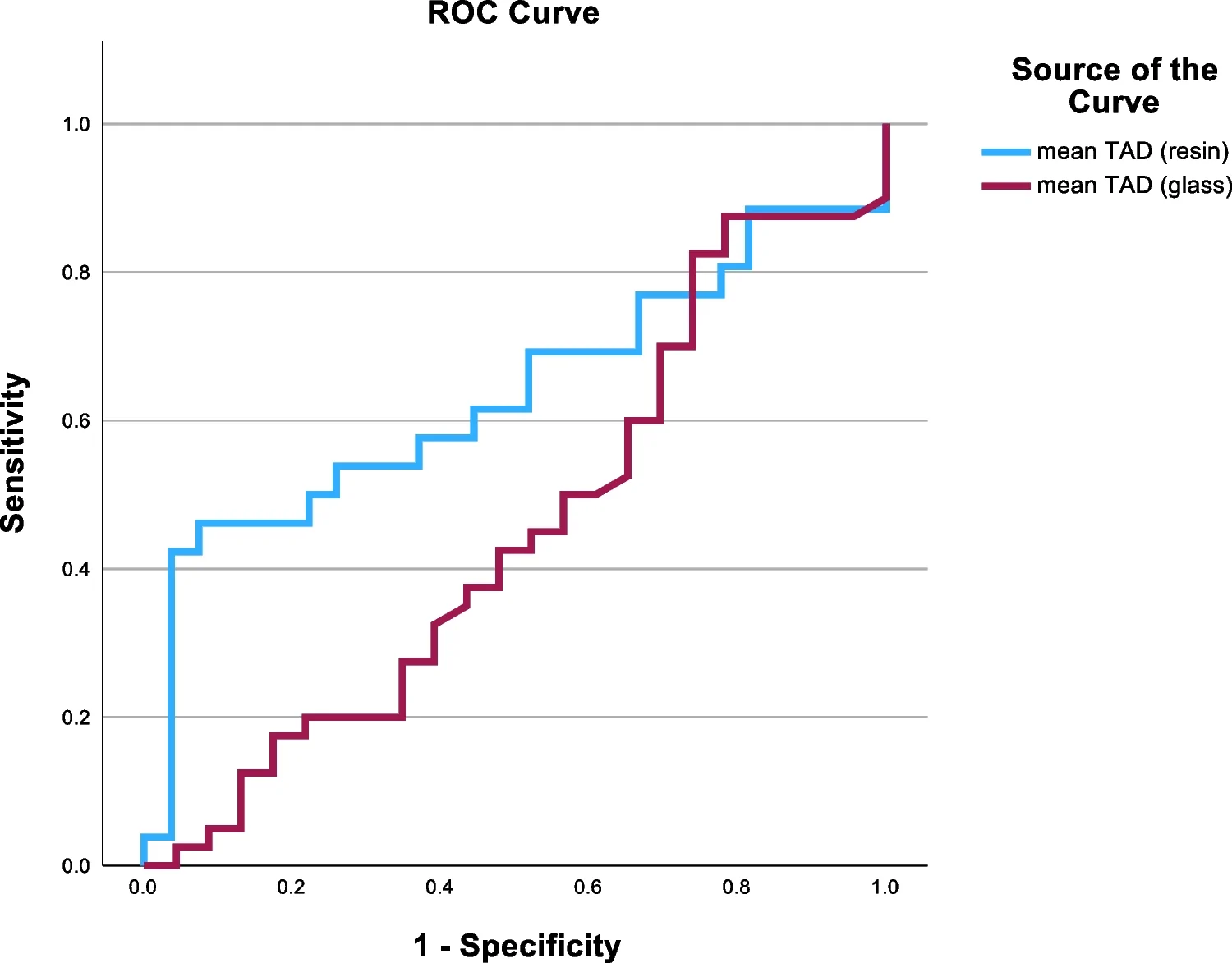
Receiver operating characteristic (ROC) curve demonstrating the predictive performance of tumor absorbed dose (TAD) for objective response (CR + PR) in patients treated with resin and glass microspheres using Y-90 PET–based dosimetry

In analyses evaluating the association between mean TAD and tumor response according to posttreatment dosimetry modality and microsphere type, a significant relationship was observed in the resin microsphere group (46 sessions) using Y-90 PET–based dosimetry (AUC 0.693, p = 0.028). Responders demonstrated significantly higher TAD values compared with nonresponders in the resin microsphere subgroup evaluated with Y-90 PET–based dosimetry (median 320 vs 166 Gy, p = 0.008) (Table 2, Fig. 2). Optimal cut-off for mean TAD in this group is calculated as 298 Gy with 55% sensitivity and 91% specificity (Fig. 3).

**Table 2:** Distribution of means and medians of TADs of responder and nonresponder lesions treated with resin microspheres evaluated with Y-90 PET–based dosimetry

**Group**	**n**	**Mean TAD (Gy)**	**Median TAD (Gy)**	**SD**
**Responder (CR+PR)**	19	358.1	320.0	237.4
**Nonresponder (SD+PD)**	27	179.6	166.0	138.8

### Dose-toxicity evaluation

Pretreatment albumin–bilirubin (ALBI) grades were 1 and 2 in 158 (96%) and 5 (4%) treatments, respectively. After treatment, ALBI grades were 1 in 146 treatments (90%), 2 in 16 (9%), and 3 in 1 (1%). An increase in ALBI grade was observed in 15 treatments (9%) from grade 1 to grade 2 and in 1 patient (1%) from grade 1 to grade 3. ALBI upgrades were observed in 8 treatments with resin and 8 treatments with glass.

In resin treatments mean perfused liver absorbed dose (PLAD) was 60.33 ± 33.5 Gy in patients with ALBI grade worsening and 9.5 ± 4.2 Gy in those without ALBI worsening (p = 0.2). In contrast, mean whole-liver absorbed dose (WLAD) was significantly higher in patients with ALBI grade worsening compared with those without (45.0 ± 21.9 Gy vs 8.2 ± 7.7 Gy, p = 0.04). In glass treatments mean perfused liver absorbed dose (PLAD) was 102.23 ± 48.5 Gy in patients with ALBI grade worsening and 33.5 ± 4.3 Gy in those without ALBI worsening (p = 0.1). Similar to resin treatments, mean whole-liver absorbed dose (WLAD) was significantly higher in patients with ALBI grade worsening compared with those without in glass treatments (98.4 ± 48.9 Gy vs 14.6 ± 7.2 Gy, p = 0.03).

One patient developed grade 2 hepatotoxicity and irreversible hepatic failure 10 months after a second TARE session due to tumor progression. In addition, cholangitis occurred in 1 patient and gastritis in 3 patients following TARE.

### Survival

**Fig. 4:**
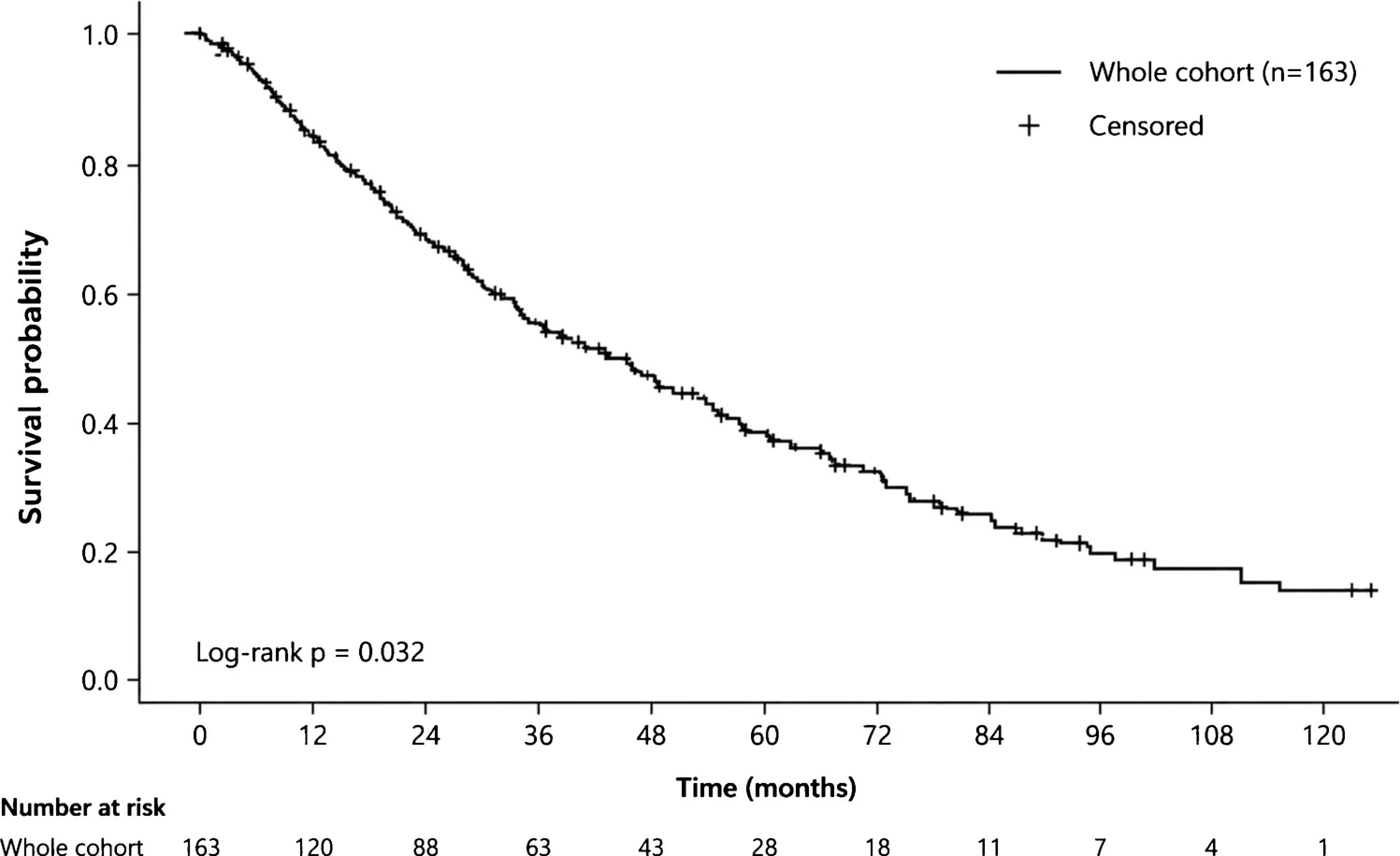
Survival curve of whole patient group

In the overall survival (OS) analysis of 180 patients with a median follow-up of 13.3 months (range 7–81), 128 deaths occurred (Fig. 4). The median OS for the entire cohort was 16.6 months (12.1–21.1, 95%CI).

**Fig. 5:**
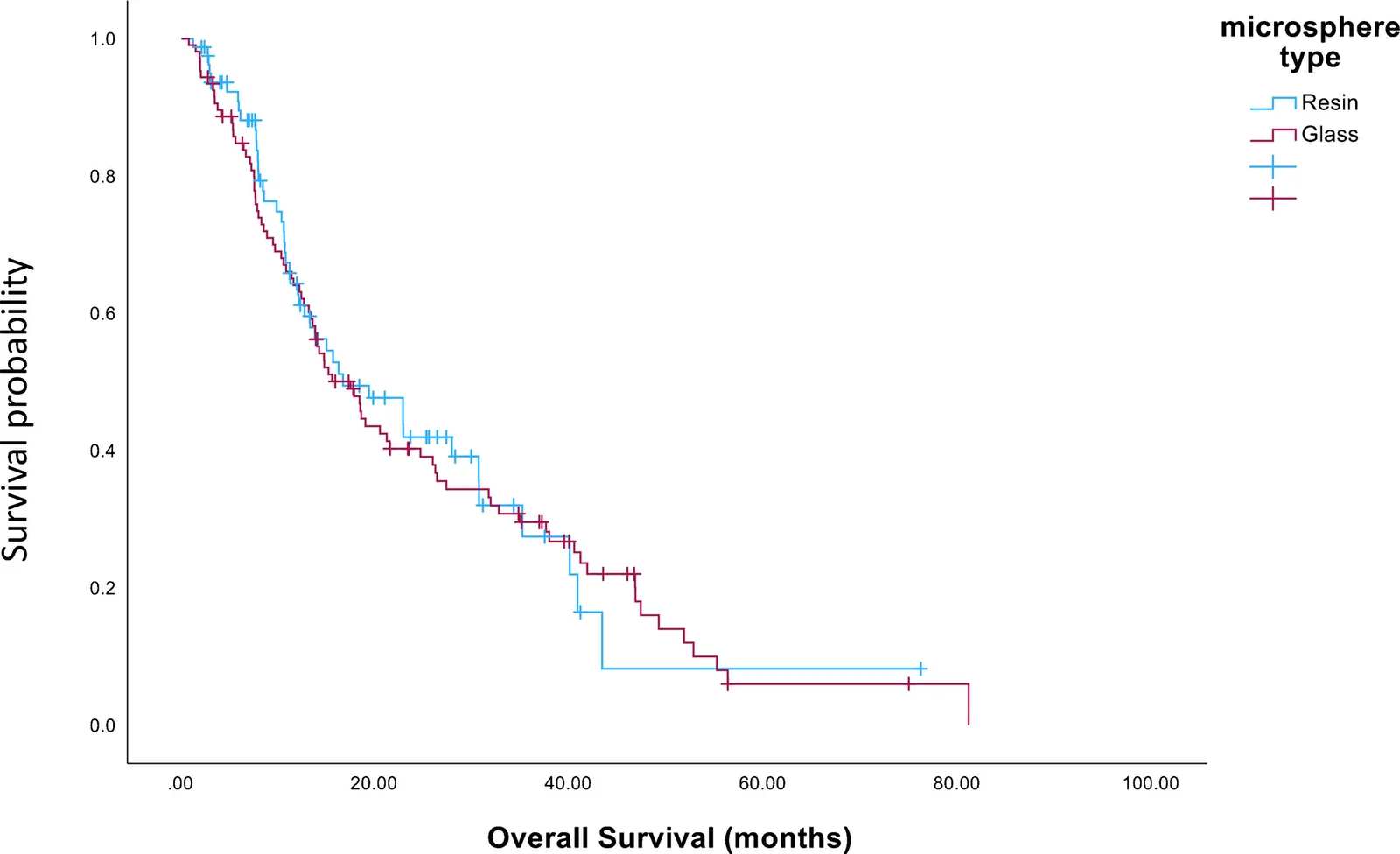
OS curves of patients treated with both microsphere types

**Fig. 6:**
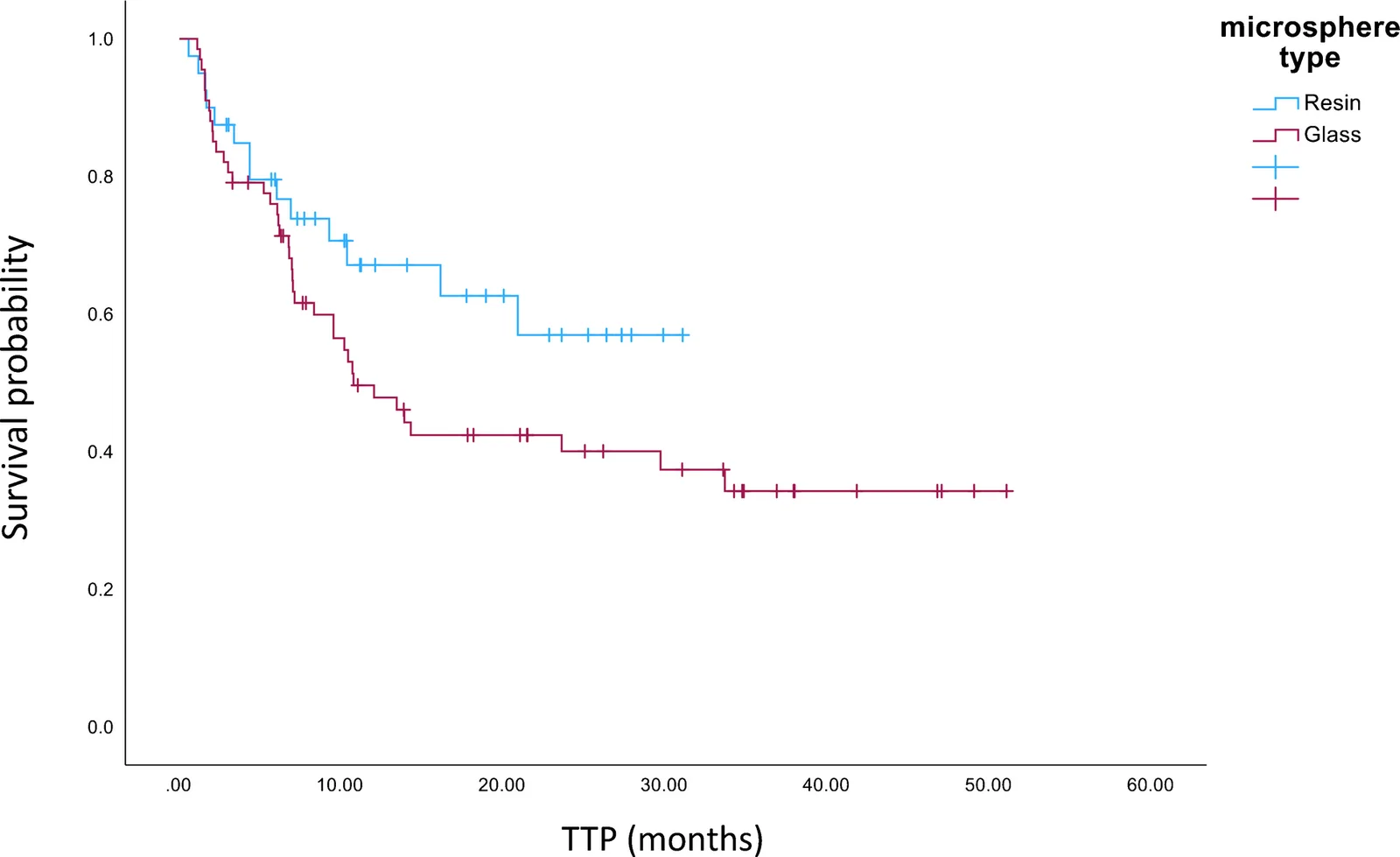
TTP survival curves of patients treated with both microsphere types

Median OS did not differ between patients treated with glass versus resin microspheres [17.4 (13.2–21.5, 95%CI) vs 16.6 (7.7–25.5, 95%CI) months, p = 0.80] (Fig. 5)***.*** Similarly, among 107 patients included in time to progression (TTP) analysis, no significant difference in median TTP was observed between patients treated with glass and resin microspheres [12.1 (7.8–12.4, 95%CI) vs 14.5 (5.6- 14.4, 95%CI) months, p = 0.11]. (Fig. 6).

In Cox proportional hazards regression analyses including age, gender, histopathological subtype, response to treatment, mean TAD and mean PLAD, serum albumin and bilirubin levels as parameters, mean TAD (p = 0.028) and pretreatment serum bilirubin levels (p = 0.022) were identified as independent predictors of overall survival in the resin microsphere subgroup.

## Discussion

Clinical experience with TARE in intrahepatic cholangiocarcinoma (ICC) remains limited compared with hepatocellular carcinoma (HCC), largely due to the lower incidence of ICC. Therefore, in this retrospective multicenter cohort, we aimed to present real-world data from a large collaborative setting. Most early data on TARE in ICC have originated from single-center retrospective series with small patient numbers, resulting in objective response rates ranging from 0 to 36% and overall survival (OS) between 8.7 and 32.3 months [13]. The major sources of heterogeneity across studies include differences in pre- and posttreatment dosimetric methodologies, variability in posttreatment imaging modalities, and interobserver variability [14–16].

To minimize these sources of variability, all dosimetric estimations in the present study were performed centrally using posttreatment Y-90 imaging. Posttreatment Y-90 SPECT and PET dosimetry were analyzed separately, and all calculations were conducted by a single experienced operator. Furthermore, TAD and PLAD thresholds differ between resin and glass microspheres, analyses were performed separately for each microsphere type.

Among the largest available datasets, the Radiation-Emitting SIR-Spheres in Non-Resectable (RESiN) registry, which included 27 centers, reported median OS and TTP of 14.0 months and 5.8 months, respectively [17]. In the phase II single-arm MISPHEC trial, which combined TARE with gemcitabine–cisplatin chemotherapy, personalized dosimetry targeting ≥ 205 Gy to the tumor yielded a median TTP of 14 months and median OS of 22 months [11]. Although these studies suggest benefit from combining TARE with systemic therapy, no evidence has demonstrated superiority of TARE over chemotherapy alone. The SIRCCA trial, designed to compare gemcitabine–cisplatin with or without TARE, was discontinued due to lack of demonstrated added value of TARE in the first-line setting [18]. In our cohort, mean OS was 25.63 ± 1.89 months (95% CI, 21.92–29.34), which is comparable to outcomes reported in first-line combination settings, despite most patients receiving TARE in a salvage context. Survival outcomes were similar between resin and glass microsphere treatments.

Optimization of posttreatment dosimetry remains a key issue in contemporary nuclear medicine practice. Both Y-90 Bremsstrahlung SPECT and Y-90 PET can be used for posttreatment dosimetry, each with distinct strengths and limitations. Although Bremsstrahlung SPECT is widely used for treatment verification, accurate quantification is challenging because of the continuous energy spectrum, absence of a photopeak, and septal penetration of high-energy photons, which degrade image resolution [19–21]. In contrast, Y-90 PET/CT may offer superior spatial resolution and more accurate dose quantification, particularly in small lesions [22–24]. These properties may improve dose–response modeling and dose–toxicity analyses. Accordingly, we evaluated SPECT- and PET-based dosimetry separately.

While strong dose–response relationships have been reported in colorectal liver metastases and HCC, in the present study a significant association between TAD and response was observed only in the resin microsphere subgroup using PET-based dosimetry.

This observation should be interpreted cautiously. The apparent association identified in the resin microsphere subgroup may partly reflect the superior quantitative accuracy and spatial resolution of Y-90 PET compared with bremsstrahlung SPECT, potentially improving the ability to detect dose–response relationships. However, because PET- and SPECT-based dosimetry were evaluated in different patients across multiple centers and treatment periods, residual confounding related to patient selection, institutional practice patterns, and other unmeasured variables cannot be excluded. Therefore, the observed association should be considered exploratory and requires confirmation in prospective studies using standardized imaging and dosimetric methodologies.

We also observed significantly higher TAD and PLAD values in PET-based compared with SPECT-based dosimetry. However, this finding cannot be generalized as a direct comparison of modalities, as this was not a head-to-head analysis in the same patients. Additionally, SPECT data were limited to a small number of centers and were derived largely from earlier treatment periods, introducing potential center- and time-related bias. For these reasons, higher estimated TAD and PLAD values observed in sessions analyzed with PET-based dosimetry; should be interpreted in the context of non-paired imaging, center-related differences, and temporal changes in practice.

Regarding safety, one patient developed grade 2 hepatotoxicity and subsequently experienced irreversible hepatic failure after a second TARE session; however, the hepatic failure was attributed to tumor progression rather than treatment-related toxicity. Reversible cholangitis and gastritis were also observed. These toxicity rates were consistent with those reported in previous series [10, 11]. We additionally evaluated changes in ALBI score as a sensitive indicator of hepatic functional reserve. ALBI worsening occurred in 9% of sessions, with grade 2 deterioration observed in only one case, and no clinically significant hepatotoxicity was observed during follow-up in these patients.

This study has several limitations inherent to its retrospective design. Loss of follow-up data led to exclusion of some patients from survival analyses. Median TTP appeared numerically close to, and in some subgroups slightly longer than, median OS. This finding reflects differences in endpoint definitions and data availability rather than a biological inconsistency. TTP analysis was restricted to patients with available progression data, whereas OS analysis included all eligible patients with survival information. In addition, deaths without documented progression were censored in the TTP analysis. Therefore, direct numerical comparison between median OS and median TTP should be interpreted with caution. Information regarding lesion volume was not consistently available across all participating centres and therefore could not be included in the present analysis. Furthermore, posttreatment Y-90 imaging methods were not fully standardized across centers, necessitating subgroup analyses with smaller patient numbers. We acknowledge that lesion size may influence quantitative recovery, particularly for Y-90 PET and bremsstrahlung SPECT imaging, because partial-volume effects become increasingly relevant in smaller lesions. Consequently, lesion volume may have acted as an unmeasured confounder in the observed dose–response analyses.

Despite these limitations, this study represents one of the largest multicenter series of ICC patients treated with TARE. Importantly, it provides comparative data on resin and glass microspheres and evaluates the dosimetric performance of different posttreatment imaging modalities.

## Conclusion

This multicenter analysis suggests that TARE may provide encouraging survival outcomes with acceptable toxicity in patients with ICC, including salvage settings. A potential dose–response association was identified in the resin microsphere subgroup evaluated with Y-90 PET-based dosimetry. Given the retrospective multicenter design and heterogeneity of imaging modalities, these findings should be considered hypothesis-generating and require confirmation in prospective studies using standardized posttreatment dosimetry.

## Data Availability

The datasets generated and analyzed during the current study are not publicly available but are available from the corresponding author on reasonable request.
